# Selective intra-operative parathyroid hormone in re-do neck exploration in parathyroidectomy: A case report

**DOI:** 10.1016/j.ijscr.2019.02.031

**Published:** 2019-02-28

**Authors:** Henry To, Gregory Otto

**Affiliations:** Department of Surgery, Lyell McEwin Hospital, Haydown Rd., Elizabeth Vale, South Australia, 5112, Australia

**Keywords:** Parathyroidectomy, Parathyroid adenoma, Intra-operative assay, Non-localised parathyroid, Case report

## Abstract

**Introduction:**

Intra-operative parathyroid hormone (I-OPTH) levels are an adjunct used in parathyroid surgery which provides assurance that parathyroidectomy surgery is complete and has removed the hypersecreting gland.

**Presentation of case:**

A 78 year old female with no localisation on pre-operative imaging and had previous hemithyroidectomy. I-OPTH was used to guide the side and timing for completion of exploration, factors which reduced operative time, operative cost and avoided potential complications.

**Discussion:**

Routine and selective I-OPTH may be used to guide parathyroidectomy, and this case highlights the methodology of use in a selective challenging case. Appropriate application and interpretation of results must be achieved to ensure adequate completion of surgery and cure for the patient.

**Conclusion:**

Selective use of I-OPTH guides parathyroidectomy in challenging cases, and can be time and cost-effective when used appropriately.

## Highlights

• Intra-operative parathyroid hormone (I-OPTH) measurement is essential in challenging cases, where there has not been localisation of the hyperfunction gland/s and/or redo neck surgery. The difficulty of the case is compounded by these factors.
• Appropriate methodology is required to perform and interpret I-OPTH, which includes specific and consistent location of sampling (ipsilateral internal jugular vein), and application of verified protocols.
• Appropriate use of I-OPTH can reduce operative time, cost and complications.

## 1 Introduction

Intra-operative parathyroid hormone (I-OPTH) may guide decision making in parathyroid surgery. Surgical resection is the only curative treatment for primary hyperparathyroidism, and identification of abnormal glands are the key to successful surgical management [1]. PTH has a predictable and short half-life, which is expected in reduce by half or more in minutes after removal of all hypersecreting glands [2]. Therefore, I-OPTH has been used as an adjunct to pre-operative imaging which, when it results to near normal range, it can provide assurance that a focused parathyroidectomy has been adequately performed and the operation is complete. Published data show that this a highly accurate technique with a 98–100% positive predictive value for a single parathyroid adenoma [3].

Our institute has reviewed local data regarding its routine use via a duel criteria protocol [4] with good outcomes in an Australian setting. I-OPTH requires on-site biochemical analysis to achieve an appropriately rapid turnaround time using direct delivery of samples from the operating theatre and a rapid immmunochemiluminescence assay. Samples were taken directly from the ipsilateral internal jugular vein pre- and 10 min post-gland excision. Results were telephoned directly to the operating theatre, often within 20 min of blood sample. Following the Miami criterion [5], a PTH reduction of greater than 50% after 10 min is an appropriate reduction and reflective of the removal of the hyperactive gland.

I-OPTH has been recommended for routine and/or selective use depending on institute equipment availability and expertise. Consensus from the American and European society of endocrine surgeons recommend I-OPTH use in challenging cases, such as when pre-operative localisation is not concordant, or in re-operative neck surgery. This case report outlines a particularly challenging case of where preoperative localisation was uncertain in a patient with previous neck surgery. It highlights the value of I-OPTH as decision making tool in selected cases of parathyroid surgery. The case report has been prepared in line with the SCARE criteria [6].

## 2 Presentation of case

A 78 year-old female had a previous right hemithyroidectomy 12 years ago. Unfortunately, the histopathology from the previous operation was not available, but it was presumed that no parathyroid glands were removed in this operation. She was subsequently diagnosed with asymptomatic hypercalcaemia (Corrected calcium 2.81 mmol/L) and primary hyperparathyroidism (PTH 21 pmol/L). Pre-operative imaging and biochemical studies (neck ultrasound, Sestamibi nuclear medicine scan, 4D computer tomography contrast neck study and bilateral percutaneous selective jugular venous sampling) failed to localise the hyperfunctioning parathyroid gland.

**Fig. 1:**
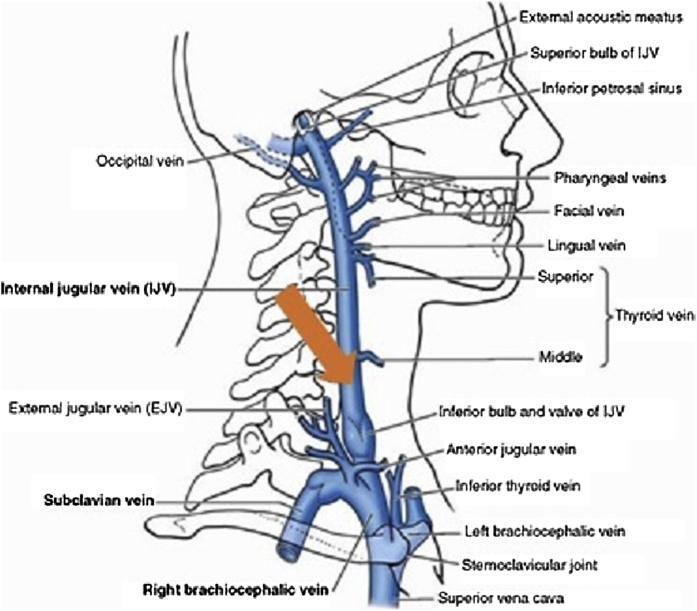
Consistent access point for sampling internal jugular vein (orange arrow) for intra-operative parathyroid hormone level, distal to middle thyroid vein [21].

As re-operative neck exploration carried a high risk of recurrent laryngeal nerve injury, lateralisation was therefore attempted via intra-operative bilateral jugular venous rapid PTH sampling (PTH STAT, Roche 2016). Samples were taken from the same junctional location bilaterally (Fig. 1), and pre-gland excision I-OPTH was higher on the left side (104.3 pmol/L left vs. 43.4 pmol/L right). A left unilateral exploration could not identify enlarged parathyroid tissue after thorough search in all normal and ectopic locations. Frozen sections were unable to confirm parathyroid gland in the excised tissue.

**Fig. 2:**
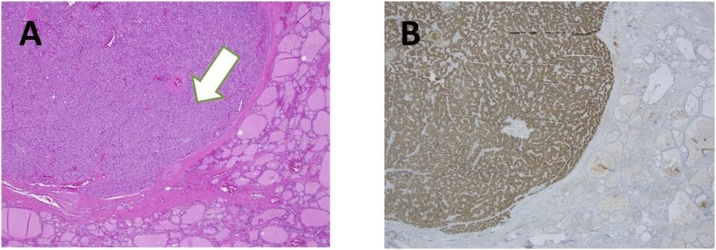
A) 100× Haematoxylin and Eosin Stain with parathyroid tissue (marked with arrow) and normal thyroid follicles, representing an intra-thyroidal, enlarged parathyroid adenoma; B) 100× PTH immunohistochemical stain of same area, with parathyroid tissue staining brown.

Finally, a left hemithyroidectomy was completed due to a presumed intra-thyroidal hyperfunctioning gland. Post I-OPTH taken from only the left internal jugular vein at 10 min appropriately reduced to 9 pmol/L, and therefore a right exploration (which would have been in scarred tissue) was not performed. Post-operative PTH at Day 1 and Day 7 were normal at 4 pmol/L and 3 pmol/L respectively and calcium also return to normal. She did not have any complications and normal voice at Day 1 and Day 7. Histopathology subsequently identified an intra-thyroidal parathyroid adenoma in normal thyroid tissue (Fig. 2). She was prescribed lifelong thyroxine and did not require calcium supplementation.

## 3 Discussion

This case highlights the value of selective use of I-OPTH in a challenging case. I-OPTH is used as an adjunct to confirm that the hypersecreting parathyroid gland/s have been removed. In this case report, the patient had a non-localised, ectopically located parathyroid adenoma where the risk of complications is greater due to previous neck surgery. Therefore, I-OPTH can be useful in such situations to guide operative management and avoid complications. In addition, it may also be useful in routine focussed parathyroidectomy.

Focused parathyroid exploration is a safe and effective procedure with high cure rates in experienced centres. It has been reported to have shorter operating time, shorter hospital stay, lower hospital costs, and reduced incidence of hypocalcaemia and other complications [7]. Effective and accurate pre-operative localisation with intra-operative histopathological confirmation of parathyroid tissue are keys to the success of the focussed approach. As an additional adjunct, I-OPTH use has been reported to improve success rate of focussed parathyroidectomy to 98% or above [2]. On the contrary, in the absence of I-OPTH, there have been reports of failure rates of up to 10% [8], even with concordant, dual modality pre-operative localisation. This may be due to, in part, by multi-gland disease, which has a reported incidence ranging from 2.4 to 34%. With the addition of I-OPTH to focussed parathyroidectomy, large studies have showed equivalence in long-term outcomes when compared with bilateral exploration [9]. In addition, a large retrospective study highlights that a large percentage decrease in serial I-OPTH values is the only protective factor against recurrence [10], thus its use may also guide the approach to follow up. For the reasons described, I-OPTH is widely used in some countries as an adjunct to determine the completion of surgery [11].

Alternatives to I-OPTH are not yet in routine clinical practice and require specialised expertise or equipment. Indocyanine green fluorescence guidance [12] or laser speckled contrast imaging [13] currently require specialist agents or equipment. Parathyroid gland aspiration may confirm hypersecreting tissue, but requires appropriate expertise. Topical application of ligocaine to cause vasodilation of the vascularised parathyroid gland may have potential unwanted side effects on the exposed recurrent laryngeal nerve. Hence, I-OPTH is a current and well-recognised method for hypersecreting gland identification which is safe and easy to employ.

However, I-OPTH has been criticised as not cost effective nor value adding, particularly in routine, localised cases [14]. Some studies report that I-OPTH used with single or dual modality parathyroid adenoma localisation have a marginal improvement in success rates of only 1 or 2% [15]. In addition, incorrect interpretation of results or false positives may lead to unnecessary neck exploration that lengthens surgical time, with increased morbidity and cost [8]. In our case, we used the Miami protocol [16] which is a validated method of interpretation, based on a percentage (rather than absolute) change and has a longer time interval for testing which ensures that there has been enough time for PTH wash out. Also, some authors also argue that compared with intra-operative frozen section, there may be longer operative time while waiting for I-OPTH results [17], thus reducing overall cost-effectiveness. Institute specific cost-effective analyses show an increased cure rate at approximately 4% additional cost [15]. Overall, its use in focussed parathyroidectomy are balanced by availability and efficiency within each institute.

In challenging cases, such as the one described, selective use of I-OPTH with appropriate test interpretation has been advocated as beneficial for patient care [8]. In the setting of discordant or non-localised pre-operative imaging and in this case report, bilateral I-OPTH may guide exploration to a single side. In multi-gland disease, single modality imaging (either US or sestamibi scan) have high failure rates (up to 30–40% of cases). However, the addition of I-OPTH when preoperative imaging is equivocal can improve these rates by identifying multi-gland disease [8].

I-OPTH may be selectively used in situations to avoid complications. Re-operative neck surgery carries a high risk of recurrent laryngeal nerve (RLN) injury. Avoidance of uni- or bilateral RLN injury has been reported to have significant morbidity on cost-benefit analysis [18]. This case highlighted the avoidance of re-operative neck surgery by guiding surgery to a single side. In low volume centres, routine use of I-OPTH has been suggested to act as a fail-safe or compensation for anatomical intricacies that may not be appreciated [19]. It is also viewed in these settings to maximise safety and prevent future re-exploration [8]. It has also been used in paediatric populations to limit the morbidity of bilateral exploration, anaesthetic time and risk to surrounding structures [20].

## 4 Conclusion

In conclusion, the benefits of I-OPTH in routine cases are institute specific but are generally well-established. However, its selective use is most beneficial in challenging cases such as non-localised and re-operative surgery for operative guidance, which improves safety and cost-effectiveness. Where possible and correctly utilised, I-OPTH is a good adjunct and should be used accordingly.

## Conflicts of interest

None.

## Funding

None.

## Ethical approval

Exception from ethical approval – case report only, consent from patient provided at request.

## Consent

See text in manuscript.

## Author’s contribution

Henry To: conceptualisation, methodology, software, validation, formal analysis, investigation, resources, data curation, writing – original draft, writing – review and editing, visualisation, project administration, funding acquisition.

Greg Otto: conceptualisation, methodology, writing – review and editing, supervision.

## Registration of research studies

Not required.

## Guarantor

Henry To and Greg Otto.

## Provenance and peer review

Not commissioned externally peer reviewed.
